# The influence of distance to perennial surface water on ant communities in Mopane woodlands, northern Botswana

**DOI:** 10.1002/ece3.4692

**Published:** 2018-12-27

**Authors:** Fredrik Dalerum, Tarryn Anne Retief, Carl Peter Havemann, Christian T. Chimimba, Berndt Janse van Rensburg

**Affiliations:** ^1^ Research Unit of Biodiversity (UMIB, UO‐PA‐CSIC) University of Oviedo Mieres Spain; ^2^ Department of Zoology and Entomology, Mammal Research Institute (MRI) University of Pretoria Hatfield South Africa; ^3^ Department of Zoology Stockholm University Stockholm Sweden; ^4^ Department of Zoology and Entomology University of Pretoria Hatfield South Africa; ^5^ Department of Zoology and Entomology, DST‐NRF Centre of Excellence for Invasion Biology (CIB) University of Pretoria Hatfield South Africa; ^6^ School of Biological Sciences University of Queensland St. Lucia Queensland Australia; ^7^ Department of Zoology, DST‐NRF Centre of Excellence for Invasion Biology (CIB) University of Johannesburg Auckland Park, Johannesburg South Africa

**Keywords:** community, diversity, Formicidae, gradient, modularity, nestedness

## Abstract

Studies of biodiversity along environmental gradients provide information on how ecological communities change in response to biotic and abiotic factors. For instance, distance to water is associated with several factors that shape the structure and the functioning of ecosystems at a range of spatial scales. We investigated the influence of distance to a perennial water source on ant communities in a semi‐arid savanna in northern Botswana. Ant abundance, taxonomic richness, and both alpha and beta diversity were generally higher during the wet than the dry season. However, there were strong seasonal influences on the effects of distance to water, with more pronounced effects during the wet season. While both abundance and beta diversity declined with increasing distances to water during the wet season, there was a contrasting increase in alpha diversity. There was no major effect of distance to water on taxonomic richness during either season. Beta diversity was as high across as along gradients, and we found support for modular rather than nested community structures along gradients. Our study demonstrated that small‐scale gradients in distance to water can influence several aspects of ant communities in semi‐arid savannas. However, our results also point to strong effects of small‐scale environmental variation, for instance associated with vegetation characteristics, soil properties, and plant community structure that are not directly linked to water access.

## 1 INTRODUCTION

The geographic distribution of biodiversity is strongly affected by environmental gradients (Currie, 1991; Hawkins, Diniz‐Filho, Jaramillo, & Soeller, 2007). In arid regions, the availability of surface water is critically important for many organisms. Therefore, distance to the nearest available water forms one of the most prominent local and regional environmental gradients in arid ecosystems (Gaylard, Owen‐Smith, & Redfern, 2003). Distance to the nearest available water can influence the distribution of plant and animal species both directly and indirectly. For example, improved hydrological conditions close to water sources generate direct positive effects on plant communities (Todd, 2006), which is generally positively associated with richer diversity of invertebrates as well as a higher abundance of vertebrate herbivores (Thrash, Theron, & Bothma, 1995). However, most vertebrate herbivores are forced to forage close to water because of daily water requirements (Redfern, Grant, Biggs, & Getz, 2003), which may also cause herbivore‐induced changes to plant communities that are not directly linked to hydrology (Lange, 1969). Similarly, historical fluvial processes have left catenal gradients that frequently also correspond to distance to water (Macklin et al., 2006), which may further influence species composition along distance to water gradients in ways not directly linked to water access.

Within savanna ecosystems, herbivore‐mediated changes to vegetation have been widely examined (Brits, Rooyen, & Rooyen, 2002; Gaylard et al., 2003; James, Landsberg, & Morton, 1999). Significant relationships have been found between several aspects of herbivore ecology and distance from water (Thrash et al., 1995; Western, 1975), including herbivore‐mediated effects vegetation parameters (e.g., Adler, Raff, & Lauenroth, 2001; Todd, 2006), herbivore distributions (e.g., Shannon, Mathews, Page, Parker, & Smith, 2009; Thrash et al., 1995; Western, 1975), and habitat utilization (Brits et al., 2002; Franz, Kramer‐Schadt, Kilian, Wissel, & Groeneveld, 2010; Fullman & Child, 2012; Gaugris & van Rooyen, 2010; Redfern et al., 2003). The so‐called piosphere effect (Lange, 1969), that is, an increased herbivore‐induced effects on vegetation close to water sources, has been found to be most severe up to 200–300 m of the water source (Thrash 1998). In this zone, there is often little or no herbaceous vegetation due to the trampling effect of large herbivores moving to and from the water source (Van der Schijff 1959; Thrash and Derry 1999). The intensity of herbivore‐induced changes to vegetation around water sources has been found to be most severe at the peak of the dry season, and to only extend for a small distance (approximately 500 m) into the surrounding vegetation (Thrash et al., 1995). However, biotic processes such as herbivory interact with variation in soil properties and hydrological processes, which leads to complex patterns of diversity in savannah ecosystems (Baldeck et al., 2014; Scholes & Walker, 1993).

Ants (Hymenoptera: Formicidae) form the most species‐rich and ecologically diverse group of social insects (Hölldobler & Wilson, 1990). Ants have been utilized in a wide range of ecological studies, for example, to investigate the biological consequences of urbanization (Rocha‐Ortega & Castaño‐Meneses, 2015; Ślipiński, Żmihorski, & Czechowski, 2012), fire (Andersen, Hertog, & Woinarski, 2006), habitat restoration efforts (Williams, Mulligan, Erskine, & Plowman, 2012), and rangeland utilization (Hoffmann, 2010). Habitat complexity may structure ant assemblages at local scales (Gibb & Parr, 2010), but the compositions of ant assemblages are influenced by both spatial and temporal variation in climate and resource availability (Barrow & Parr, 2008; Bishop, Robertson, Rensburg, & Parr, 2014; Hodkinson, 2005; Munyai & Foord, 2012, 2015). Effects of distance to water availability on ant communities seem mostly related to hydrological effects on microhabitats and microclimate (e.g., Levings, 1983). However, such effects are not always uniform. For instance, ant species appeared to have specific preferences for vegetation associated with different water column depths in a Florida longleaf pine savannah (Tschinkel, Murdock, King, & Kwapich, 2012), and ant activity differed between microhabitats along both temporal and spatial humidity gradients in the Amazonian rainforests (Kaspari & Weiser, 2000).

Biodiversity is often decomposed into alpha (α), beta (β), and gamma (γ) components (Jost, 2007), where gamma diversity describes regional diversity which is further partitioned into alpha and beta components which describes within‐ and between‐group diversity (Ellison, 2010). Alpha diversity describes the diversity within a specific location, and beta diversity describes the variability in community composition among sampling units for a given area (Anderson, Ellingsen, & McArdle, 2006; Whittaker, 1960). Beta diversity can therefore be essential for understanding the processes underlying the formation and evolution of ecological systems (Legendre & De Cáceres, 2013). However, while measurements of alpha, beta, and gamma diversity are useful for assessing variation within and among ecological communities, they do not fully capture the way species structure themselves across time and space (but see Anderson et al., 2011 for alternative interpretations).

Spatial and temporal patterns of community structures may however be critically important for ecosystem functioning (Loreau et al., 2001), and form the basis for landscape level (i.e., gamma) diversity (Whittaker, Willis, & Field, 2001). In particular, nested and modular structures have been suggested as particularly important for ecosystem function and stability (Reiss, Bridle, Montoya, & Woodward, 2009). Nestedness describes a non‐random species distribution in which the least widely distributed species occupy areas that are also occupied by species with wider distributions (Patterson & Atmar, 1986). Species‐rich locations therefore contain unique species, and consequently, species‐poor locations do not contribute to the overall taxonomic richness of an area (Ślipiński et al., 2012). The concept of modularity has received a great deal of attention in recent years in the study of trophic networks (reviewed in Miranda, Parrini, & Dalerum, 2013), but the concept is rarely applied in a spatial context (Dalerum, Vries, Pirk, & Cameron, 2017). In a spatial context, modular structures of species distributions would suggest locally distinct species assemblages in which a given species primarily would co‐exist only with other species from its specific local assemblage. Therefore, in contrast to nested structures, each local assemblage would contribute to the overall taxonomic richness and diversity of an area.

In this study, we investigated the influence of distance to a perennial water source on ant abundance, alpha and beta diversity, and community composition in a Mopane woodland in northern Botswana. To our knowledge, this is the first ant study in this region, and there is a general lack of studies examining the importance of distance to permanent water in structuring invertebrate communities in arid savannah ecosystems. We predicted that close proximity to water would be associated with increased ant abundance, taxonomic richness and diversity, due to higher productivity and microhabitat complexity. We also expected that ant communities would structure in a nested pattern along an environmental gradient away from the water, that is, that the taxonomically poorer communities outside the zone that may be positively influenced by perennial water would be subsets of the taxonomically richer communities closest to the water. We regard this to be an appropriate prediction in the absence of specific information regarding habitat specialization for most ant species, since it assumes that the species in the poorest environments also are present in more favorable ones. We highlight that we do not necessarily expect any effects of distance to water to be directly caused by water access, but rather by the structure and composition of habitat variables relevant to ants. Such habitat variability is likely influenced by a range of processes, including herbivory‐ and soil‐induced effects of vegetation composition and structure. However, since we regarded it to be most germane to first quantify the patterns of spatial variation in ant communities before evaluating potential causes underlying such patterns (Dalerum, 2012), we have here not included any direct correlates to diversity along the gradient, such as soil type, vegetation structure, or grazing pressure. We regard such an analysis to be most efficient once patterns of ant communities have been adequately identified. Finally, we also predicted that ants would be more active and abundant during the wet season, and hence that they through sample processes would show higher taxonomic richness and be more diverse during the wet season than during the dry, particularly in diversity poor sites. This would result in a spatial homogenization of ant diversity during the wet season.

## 2 MATERIALS AND METHODS

### 2.1 Study area

**Figure 1:**
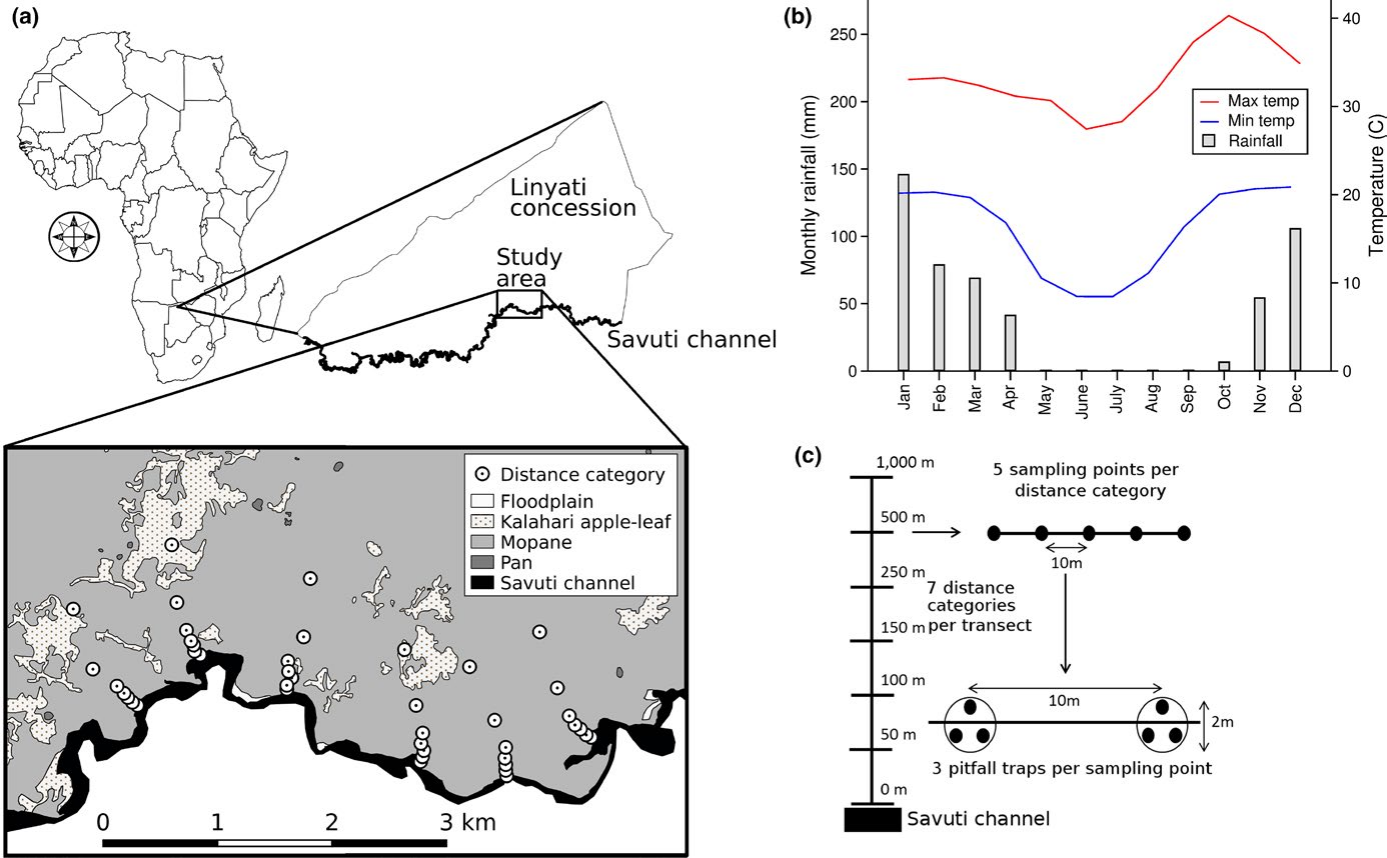
Map showing the Linyanti concession of northern Botswana including the study area and the selected transects (a), climate data 2012–2015 averaged across three sites within the Linyanti concession (b), and a schematic diagram depicting the survey design (c). We sampled ant communities along six transects placed perpendicularly to the perennial water source and extending into Mopane woodlands; each transect had a length of 1 km and contained five sampling points at each of seven distances from water (0, 50, 100, 150, 250, 500, and 1,000 m). At each sampling point, we placed three pitfall traps within a 2 m radius

We conducted the study around the perennial Savuti channel in the Linyanti concession of northern Botswana (between S18°16′48″/E23°53′03″ and S18°42′05/E23°37′07″: Figure 1a). The region has a semi‐arid climate characterized by strongly seasonal rainfall. The study area falls within a summer‐rainfall zone and had a mean precipitation of 502 mm (±1 *SD* 94 mm) per year during 2012–2015. The wet season mainly occur from November through March and the dry season from April to October (McCarthy, Bloem, & Larkin, 1998). Average monthly rainfall 2012–2015 for the study area was 91 mm for the wet season (November–March) and 7 mm for the dry (April–October), with average maximum and minimum temperatures of 34 and 20°C for the wet and 32 and 13°C for the dry season (Figure 1b). Flood levels during the wet seasons of 2009/2012 reached a maximum of 70 cm above dry season baselines. The area is predominantly flat and dominated by Mopane (*Colophospermum mopane*) woodlands intersected by woodlands characterized by Kalahari apple‐leaf (*Philenoptera nelsi*). Seasonal pans, that is, flat areas with standing open water, occur throughout the concession. Together with the permanent water in the Linyanti River and Savuti Channel, they result in a high game density. Significant populations of elephants and other herbivores are found along the Linyanti River and Savuti Channel in the dry season when water resources are restricted elsewhere, and results in the Linyanti concession having one of the highest elephant densities in Africa (Barnes et al., 1999).

### 2.2 Survey design

We sampled ant communities using pitfall traps placed along six transects located perpendicularly to the perennial water source and extending into Mopane woodlands (Figure 1a). A minimum distance of 750 m separated different transects. Each transect had a length of 1 km with five sampling points located at seven distances from water (0, 50, 100, 150, 250, 500, and 1,000 m, Figure 1c). At each sampling point, we placed three pitfall traps within a 2 m radius. This sampling design thus enabled us to evaluate variation in ant diversity at multiple spatial scales, which is important for appropriate identification of biodiversity patterns (Dalerum et al., 2017). Pitfall trapping is a commonly used method to evaluate ant community structure, and in areas where leaf litter is not deep, the quality of this method in terms of its utility for sampling ant abundance ranks among the highest when compared to different methods typically used for sampling ants (Dunn, Guenard, Weiser, & Sanders, 2010).

All transect were sampled once during each season (wet season: 20 February–13 April 2012; dry season: 23 June–29 July 2012), although for logistical reasons some wet season sampling continued into April. Rainfall during these trapping periods was 83 mm for the wet and 9 mm for the dry season. The pitfall traps remained in the field for a total period of five trap nights, although we serviced the traps after the third trap night by replacing the initial trap with a new one to prevent trap loss during the wet season due to flooding. However, to maintain a consistent protocol, we replicated the procedure during the dry season. The traps were plastic containers with a diameter of 5.9 cm filled with 30% propylene glycol in water. Pitfall traps were covered with 25 cm diameter plastic rain lids which were placed at a minimum of 10 cm above the traps. For consistency, we used the rain lids in both wet and dry seasons. The pitfall traps were placed at the same location during both sampling periods.

### 2.3 Ant identification and quantification

Because of the uncertain taxonomic status of many ant species, we grouped ants into morphospecies (e.g., Rocha‐Ortega & Castaño‐Meneses, 2015), which are effective surrogates for species in environmental monitoring and conservation (Oliver & Beattie, 1996). Ants were identified into genera using online identification keys (www.antsofafrica.org; www.antweb.org). We based our morphospecies definitions on several characters which are known to be important at the species level (Parr & Chown, 2001), including the shape, width, and length of the head; the shape and length of mandibles; shape, size, and location of the eyes; shape and contouring of the alitrunk; the presence, length, location, and density of hairs; location, shape, and size of spines; length of the scape. Taxonomy followed Fisher and Bolton (2016). Ant specimens were identified by staff trained in ant identification and are stored at the permanent ant collection at the Department of Zoology and Entomology, University of Pretoria.

### 2.4 Data analyses

We quantified abundance as the number of individual ants per trap for each trapping period, taxonomic richness as the number of morphospecies per trap, and alpha diversity as the effective number of morphospecies in each trap, that is, the exponent of the Shannon–Wiener index calculated on the relative number of morphospecies captured in each trap (Hill, 1973). We quantified beta diversity as the average distance in taxonomic space from individual traps to the centroid of relevant spatial groupings (Anderson et al., 2006). Distances were calculated in a multivariate space which dimensionality was defined by the number of morphospecies. The value along each dimensional axis was the number individuals of that morphospecies in each trap. We calculated this metric in several steps and for four spatial scales: within transects (i.e., along each gradient of distance to water), within distance categories (i.e., specifying beta diversity at specific distances along the gradient of distance to water), within distance categories and transects (i.e., specifying at beta diversity at specific points along the gradient of distance to water within a transect), and within sample points (i.e., quantifying sampling variation). We first created a pair wise distance matrix of the values in all traps using a modified Gower dissimilarity, which explicitly weight an order‐of‐magnitude change in abundance the same as a change in species composition (Anderson et al., 2006). We then transformed these distances into a principal coordinate space which embeds the original distances within a Euclidean space regardless of the nature of the original distance (Anderson et al., 2006). Finally, we calculated the Euclidean distances between each trap and centroids specific for our spatial scales. For the transect scale, we used the centroid of each transect; for the distance category scale, we used the centroid of each distance category pooled across transects; for distance category within transects scale, we used the centroid of each distance category for each transect; and for sample point scale, we used the centroid of each sample point. We calculated separate distances for each season for all spatial scales.

We used generalized linear mixed models (GLMMs) to assess the effect of distance to water on ant abundance and taxonomic richness, and linear mixed models (LMMs) to assess the effects on alpha and beta diversity. In the models on abundance, taxonomic richness and alpha diversity, we used raw number of ants per trap, raw number of morphospecies per trap, and the effective species richness per trap as response variables, and fitted distance to perennial water (fitted as a discrete factor with the distance categories as factor levels), season and their two‐way interaction as fixed effects. We fitted three models on beta diversity. In the first, we used distances to relevant centroids as the response variable and fitted spatial scale of the centroid (i.e., along transects, within distance categories, within distance categories within transects, and within sample points), season and their two‐way interaction as fixed effects. We also fitted two additional models to explicitly evaluate the influence of season and distance to water on beta diversity across and within transects, one using only distances calculated within distance categories but across transects and one using only distances calculated within both distance categories and transects. In these models, we used distance category, season and their two‐way interaction as fixed effects. For all models, we added sample point nested within distance category and transect as a random effect structure to adjust for the spatial grouping of sampling points. We fitted the models on abundance and on taxonomic richness using a Poisson error family with a log link function. We evaluated fixed terms using sequential likelihood ratio tests (Zuur, Ieno, Walker, Saveliev, & Smith, 2009). Within each season, we compared the values of each distance category to the one adjacent to the water using planned contrasts on least square means (Lenth, 2016), with the alpha levels adjusted for multiple comparisons according to Benjamini and Hochberg (1995).

We adapted methods from mathematical graph theory to evaluate spatial nestedness and modularity (Dalerum et al., 2017). We based both of these analyses on interaction matrices with distance classes as columns, morphospecies as rows and the pooled number of individuals trapped within each distance class and for each morphospecies as cell values. We made one matrix for each transect and season. We quantified the nestedness of the spatial distribution of ant communities within each of the six transects using a Weighted‐Interaction Nestedness Estimator (WINE) (Galeano, Pastor, & Iriondo, 2009). Hence, in contrast to binary methods (Ulrich, Almeida‐Neto, & Gotelli, 2009), it includes a measure of abundance. The value of the estimator approaches zero when the weighted‐interaction nestedness (WIN) of the original data matrix is close to the average WIN of the equivalent random matrices (Galeano et al., 2009). Negative WINE values therefore indicate anti‐nestedness, that is, that the composition of a community is less nested than random predictions. We quantified the taxonomic modularity of the spatial distribution of ant communities within each of the six transects with an index of modularity for weighted networks using the QuaBiMO algorithm (Dormann & Strauss, 2014).

For both nestedness and modularity, we evaluated if the observed values differed from random expectations by comparing them to a reference distribution derived from a null model constrained to retain both the column and row margin totals of the observed matrices (Dormann, Fründ, Blüthgen, & Gruber, 2009). We constructed 1,000 random matrices for each of the six transects and for each season. To compare the nestedness and modularity between the wet and the dry season, we subtracted the observed values from each of the null model values (Manly, 1997), and compared these differences using a two‐sample permutation test.

We calculated the Shannon indices using Primer software version 5.2 (Clarke & Warwick, 2001), and conducted all other data analyses in the R version 3.3.2 for Linux (https://www.r-project.org), using the contributed packages bipartite (Dormann et al., 2009), coin (Hothorn, Hornik, Wiel, & Zeileis, 2008), lme4 (Bates, Maechler, Bolker, & Walker, 2015), lsmeans (Lenth, 2016), MASS (Venables & Ripley, 2002), and vegan (Oksanen et al., 2013).

## 3 RESULTS

**Figure 2:**
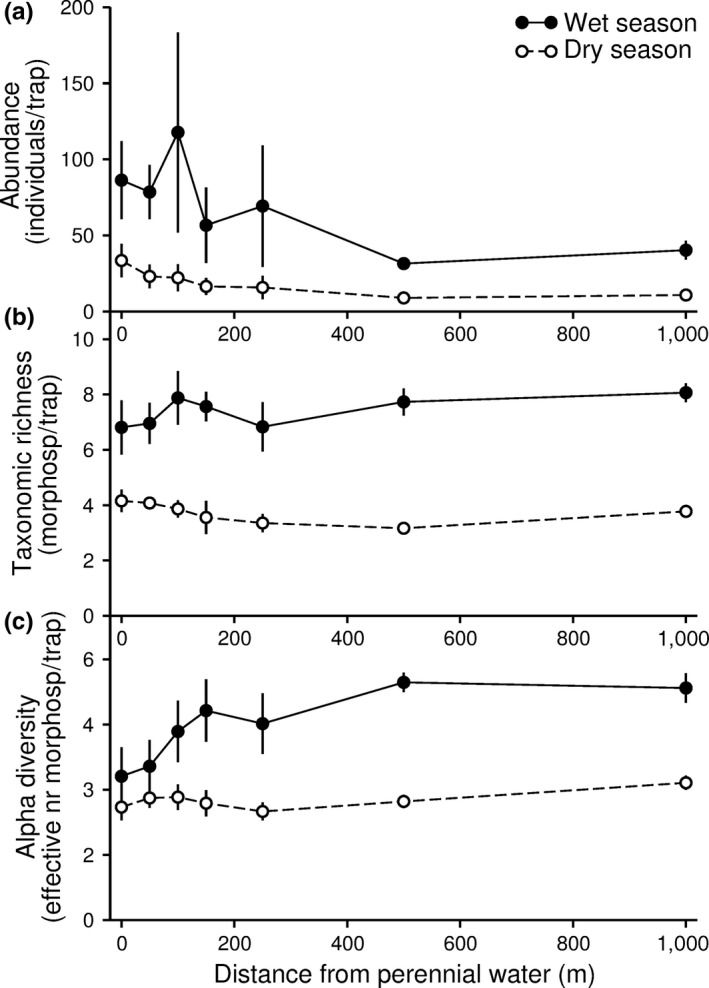
Ant abundance (estimated as number of ants per trap: a), taxonomic richness (estimated as number of ant morphospecies per trap: b), and alpha diversity (estimated as effective number of ant morphospecies per trap: c) at different distances from perennial water during the dry and wet seasons in the Linyanti concession of northern Botswana. The figure presents mean ± *SE*

**Figure 3:**
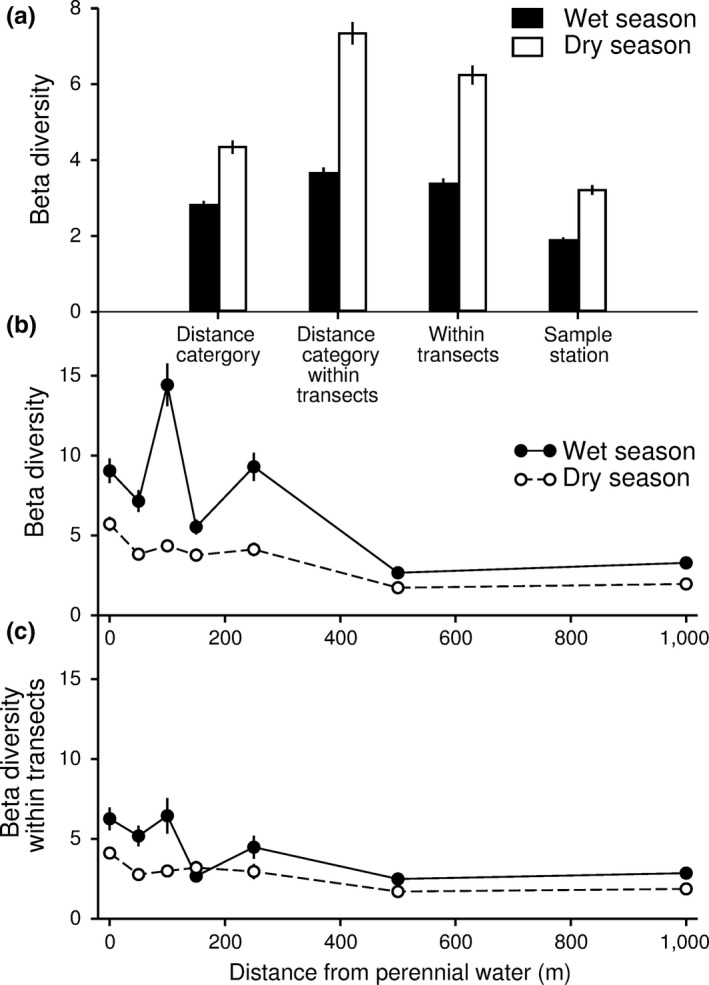
Ant beta diversity measured as distance to centroids at four spatial scales (including distance from perennial water pooled across transects, distance form perennial water within transects, along transects, and within sample station) during the wet and dry season (a), as well as beta diversity calculated across (b) and within (c) transects at different distances from perennial water during dry and wet seasons in northern Botswana. The figure presents mean ± *SE*

We identified a total of 55,032 ants to 29 genera and 149 morphospecies (Supporting Information Table A1). There were significant interaction effects of distance to water and season on ant abundance (*χ* ^2^ = 550, *df* = 6, *p* > 0.001), taxonomic richness (*χ* ^2^ = 25.2, *df* = 6, *p* > 0.001), and alpha diversity (quantified as the effective number of morphospecies, *χ* ^2^ = 81.3, *df* = 6, *p* > 0.001). There were also significant interaction effects of distance to water and season on beta diversity calculated both across (*χ* ^2^ = 76.6, *df* = 3, *p* > 0.001) and within (*χ* ^2^ = 19.5, *df* = 3, *p* > 0.001) transects. However, across all distances to water, there were consistently higher ant abundances, higher taxonomic richness, as well as higher alpha and beta diversity during the wet than the dry season (Figures 2 and 3).

Ant abundances generally declined with increasing distance from water across both seasons (Figure 2a). During the dry season, distance categories 100 m through to 1,000 m had significantly lower ant abundance than the 0 m distance category, whereas only the 500 m distance category differed significantly from the 0 m category during the wet season (Table 1). There were only weak effects of distance to water on ant taxonomic richness, with trends for declines with increasing distances from water during the dry season and contrasting increases with increasing distances from water during the wet season (Figure 2b). No distance category differed significantly in taxonomic richness from the 0 m category during either of the seasons, although there was a trend for the 500 m to have lower taxonomic richness that the 0 m category during the dry season (Table 1). The effective number of morphospecies did not decline with increasing distance to water during either season (Figure 2c). No distance categories differed from the 0 m category in terms in the effective number of morphospecies during the dry season (Table 1). In contrast, there was a general increase in the effective number of morphospecies with increasing distances from water during the wet season (Figure 2c), with ant assemblages at distance categories 150 and 1,000 m having had significantly higher effective number of morphospecies than the 0 m distance category, and with a trend also for the 100 m category to have a higher effective number of morphospecies that the 0 m category (Table 1).

**Table 1:** Estimates of beta coefficients describing the differences in ant abundance (number of ants per trap), taxonomic richness (number of morphospecies per trap), and alpha diversity (effective number of morphospecies per trap) between successive distance categories from a permanent water source and the traps adjacent to the water

	Wet season			Dry season		
	*β*	*Z*	*p*_adj_	*β*	*Z*	*p*_adj_
Abundance, m						
50	−0.098	−0.339	0.882	−0.377	−1.296	0.195
100	0.011	0.036	0.971	−0.712	−2.449	0.017*
150	−0.515	−1.778	0.113	−0.805	−2.767	0.009*
250	−0.558	−1.924	0.109	−1.089	−3.741	0.001*
500	−0.869	−2.997	0.016*	−1.184	−4.057	<0.001*
1,000	−0.628	−2.167	0.091	−0.992	−3.404	0.001*
Taxonomic richness, m						
50	0.037	0.424	0.806	−0.003	−0.027	0.979
100	0.158	1.824	0.188	−0.059	−0.591	0.666
150	0.118	1.349	0.266	−0.144	−1.415	0.314
250	0.011	0.125	0.900	−0.206	−2.013	0.132
500	0.146	1.674	0.188	−0.253	−2.451	0.085
1,000	0.191	2.211	0.162	−0.073	−0.727	0.666
Alpha diversity, m						
50	0.234	0.464	0.645	0.213	0.421	0.851
100	1.034	2.048	0.058	0.225	0.446	0.851
150	1.515	3.001	0.010*	0.096	0.189	0.851
250	1.217	2.411	0.032*	−0.096	−0.190	0.851
500	2.161	4.282	0.001*	0.127	0.252	0.851
1,000	2.030	4.022	0.001*	0.557	1.103	0.851

Notes

Beta coefficients were based on generalized linear mixed models (abundance and species richness) and linear mixed models (alpha diversity) and calculated using planned contrasts on least square means.

a Statistical significance at *α* = 0.05 after adjusted for multiple comparisons following Benjamini and Hochberg (1995).

There was a significant interaction effect of spatial scale and season on beta diversity (*χ* ^2^ = 51.5, *df* = 3, *p* > 0.001). Beta diversity was considerably higher during the wet than the dry season, and beta diversity was lowest within sample points, slightly higher in distance categories within transects, but similar within transects and distance categories pooled across transects (Figure 3a). Beta diversity calculated both across (Figure 3b) and within transects (Figure 3c) showed a declining trend along the distance to water gradient for both the wet and dry seasons. When calculated across transects, beta diversity at all but the 250 m (wet season) and the 100 m (dry season) category was significantly lower than the 0 m distance category, for both the wet and dry seasons. Within transects, distance categories 150–1,000 m had lower beta diversity than the 0 m distance category during wet season, and 500–1,000 m had lower beta diversity than the 0 m distance category during the dry season (Table 2).

**Table 2:** Estimates of beta coefficients describing the differences in beta diversity between successive distance categories from a permanent water source, calculated both across and within transects, and the traps adjacent to the water

	Wet season			Dry season		
	*β*	*Z*	*p*_adj_	*β*	*Z*	*p*_adj_
Distance category, m						
50	−1.856	−2.54	0.013*	−1.903	−2.594	0.014*
100	3.682	5.041	<0.001*	5.375	−1.892	0.058
150	−3.518	−4.831	<0.001*	−3.518	−2.695	0.014*
250	0.250	0.343	0.732	0.250	−2.414	0.019*
500	−6.387	−8.769	<0.001*	−6.387	−5.497	<0.001*
1,000	−5.764	−7.914	<0.001*	−5.764	−5.149	<0.001*
Distance category within transects, m						
50	−1.044	−1.704	0.106	−1.345	−2.201	0.056
100	−0.642	−1.048	0.295	−1.133	−1.850	0.077
150	−3.593	−5.881	<0.001*	−0.925	−1.506	0.132
250	−1.782	−2.917	0.006*	−1.171	−1.889	0.077
500	−3.772	−6.174	<0.001*	−2.471	−3.973	<0.001*
1,000	−3.407	−5.576	<0.001*	−2.249	−3.672	<0.001*

Beta coefficients were based on linear mixed models and calculated using planned contrasts on least square means.

a Statistical significance at *α* = 0.05 after adjusted for multiple comparisons following Benjamini and Hochberg (1995).

Only three of the six transects had significantly greater nested patterns of species composition than expected by chance for both the wet and dry seasons (Table 3). Transects 1 and 6 had significantly higher patterns of nestedness only during the dry season, while transect 2 had a significantly higher pattern of nestedness only during the wet season. Five of the six transects were found to have statistically different nestedness values for both the wet and dry seasons with four transects having greater patterns of nestedness during the dry season. For each of the six transects, no distance category was found to be a nested subset of another distance category. The distance category which was calculated to be the most taxonomically rich distance category occurred within 100 m of the water source during both the wet and dry seasons for all six transects (Supporting Information Figure A1). The most species‐rich distance category was the same in both the wet and dry seasons for five of the six transects.

**Table 3:** Observed and expected values of nestedness and modularity of ant communities along six transects away from a perennial water source in the Linyanti concession of Botswana

	Wet season						Dry season					Seasonal differences		
	WIN			WINE	*z*‐score	*p*‐value	WIN		WINE	*z*‐score	*p*‐value		*z*‐score	*p*‐value
	Obs.		Exp.				Obs.	Exp.					Obs.	
Nestedness														
T1		0.250	0.244	0.037	0.670	0.251	0.318	0.291	0.148	2.271	0.012		0.250	<0.001
T2		0.250	0.222	0.190	2.994	0.001	0.282	0.281	0.005	0.069	0.472		0.250	<0.001
T3		0.270	0.238	0.209	3.554	<0.001	0.295	0.259	0.228	3.156	<0.001		0.270	<0.001
T4		0.286	0.262	0.135	2.461	0.007	0.312	0.287	0.131	1.930	0.027		0.286	<0.001
T5		0.225	0.205	0.141	2.463	0.007	0.299	0.266	0.206	2.877	0.002		0.225	<0.001
T6		0.196	0.202	−0.045	−0.847	0.802	0.269	0.249	0.130	1.952	0.025		0.196	<0.001

	*Q*‐value		*z*‐score	*p*‐value		*Q*‐value		*z*‐score	*p*‐value		*z*‐score	*p*‐value
	Obs.	Exp.				Obs.	Exp.				Obs.	Exp.
Modularity												
T1	0.142	0.025	24.765	<0.001		0.094	0.033	8.692	<0.001		0.142	0.025
T2	0.141	0.047	17.787	<0.001		0.349	0.079	35.462	<0.001		0.141	0.047
T3	0.149	0.039	19.238	<0.001		0.393	0.063	38.080	0		0.149	0.039
T4	0.115	0.009	44.529	0		0.139	0.031	15.679	<0.001		0.115	0.009
T5	0.092	0.024	11.095	<0.001		0.415	0.104	34.678	<0.001		0.092	0.024
T6	0.196	0.030	22.157	<0.001		0.094	0.074	1.804	0.072		0.196	0.030

Note

Expected values as well as the corresponding p‐values for the observed values were based on 1,000 random matrices with the same row and column total as the observed matrices, and the seasonal comparison based on 2‐sample permutation tests of the difference between the observed and each of the random values (e.g., D‐values) for each transect and season.

All six transects were found to be more modular than expected by chance, during both the wet and the dry seasons (Table 3), with the exception of transect 6 in the dry season. The modularity values obtained for each transect during the wet season were found to be statistically different to their corresponding dry season values, with four of the six transects having higher modularity values during the dry season. For each of the six transects, two modules were created during the wet season, with either one or two distance categories being contained within one of the modules (Supporting Information Figure A2). When two distance categories were associated in a module, they were not separated by more than 100 m. Where only one distance category was contained within one of the modules, the distance category always fell within 250 m from the water source. During the dry season, the number of modules varied from two to five. The 500 m distance category was isolated from the rest of the distance categories in five of the six transects during the dry season, with the exception of transect 4, which was the only transect where two modules contained the same combination of distance categories (i.e., the 100 m distance category being contained within one module) in both seasons.

## 4 DISCUSSION

Our study reiterates the importance of distance to water for ecological processes in savanna ecosystems (Gaylard et al., 2003), but does not provide uniform support for higher diversity close to perennial water. Although we found both higher ant abundance and beta diversity close to a perennial water source, both taxonomic richness and alpha diversity increased with increasing distances to water during the wet season. Within the Linyanti concession, the lack of rainfall in the dry season results in very low primary productivity with most of the Mopane woodlands being devoid of fresh growth and green leaves with only very sparse undergrowth. Hence, the permanent water in the Savuti Channel may allow ants to be more active in areas close to the water by altering vegetation and soil properties. However, the decline in alpha diversity during the wet season suggests that specific species became dominant when water was more accessible. Soil, moisture, and vegetation properties have previously been shown important for ant abundance and community composition (LeBrun, Plowes, & Gilber, 2012; Munyai & Foord, 2012; Ríos‐Casanova, Valiente‐Banuet, & Rico‐Gray, 2006; Wang, Strazanac, & Butler, 2001), and our results highlight that community structure along distance to water gradients is formed by complex and interacting processes, both abiotic and biotic.

For ant abundance and both alpha and beta diversity, the effects of distance to water subsided at distances of 300 m. These observations coincide with previous suggestions that a herbivore‐mediated trampling effect on vegetation, that is, the piosphere effect, may extend 300–500 m away from a water source (Thrash et al., 1995). Although the furthest distance categories of two transects fell within a different vegetation type compared to the other transects, we suggest that part of our results may point to a piosphere effect around the Savuti channel. However, this possible piosphere effect did not appear to be uniformly positive, since we found higher abundance and beta diversity but lower alpha diversity closer to the water source. Hence, we stress that further research is needed to fully evaluate how herbivore densities are interacting with other environmental factors in their influences on ant community variation at increasing distances from perennial water.

As expected, there was a seasonal variation in the effects of distance to water on all quantified aspects of ant communities. However, we found that the effects of distance to water in general were higher during the wet than the dry season. Seasonal variation in rainfall has been shown to influence invertebrate activity patterns, competitive interactions, and the availability of resources (Wolda, 1988), including in ants (Munyai & Foord, 2015). Our results suggest that rainfall increased rather than decreased the effect of distance to water on ant communities. Hence, our data did not support that increased amount of surface water during the wet season is homogenizing the spatial patterns of primary productivity and variation in vegetation structure (O'Connor, 1994). Instead, other factors, for instance related to highly local variation in temperature regimes and moisture levels (Barrow & Parr, 2008), likely influenced the observed seasonal variation in the effects of distance to perennial water. Such an interpretation would agree with previous observations that plant productivity is influenced by processes that occur on very local spatial scales (Martin & Ferrer, 2015), and reiterates recent findings that local conditions may be critical for determining both spatial and temporal variation in arthropod communities (Dalerum et al., 2017).

Although we observed a strong effect of distance to water on beta diversity, we did not find that beta diversity was higher along the distance to water gradients than between transects. In addition, beta diversity was smaller if quantified at very local scales. These results further point to local conditions as important for shaping ant communities in this environment. Soil and vegetation types are known to have an overriding influence on ant community composition when compared to other factors such as grazing pressure (Gibb & Parr, 2010). Soil deposits along the banks of a river can alter soil nutrient availability within the vicinity of water courses. River systems are known to shift course over time due to changes in water flow and hydrology, and these shifts may result in altered soil properties which may occur in very patchy distributions (Deil, 2005). Our results highlight that factors related to such geological processes may have been very influential for shaping ant communities in this ecosystem.

We observed nested patterns of ant community composition only along half of the transects, and we did not observe consistent seasonal differences in the level of nestedness. Instead, we found consistent modularity along all transects, and the majority of the transects were more modular during the dry than the wet season. These observations highlight that the effects we observed in terms of variation in ant abundance, species richness, and diversity over different distances from water seem to have been caused by distinct species combinations at each local community. We suggest that this observation lends further support to our interpretation that local characteristics have strong effects on local ant communities, and we suggest that further research is directed to quantify which environmental variables are associated with variations in ant communities and different spatial scales.

## 5 CONCLUSIONS

We demonstrated that small‐scale gradients in distance to water can influence several aspects of ant communities in semi‐arid savannas. However, although we found higher ant abundance and beta diversity in the vicinity of a perennial water source, we found limited effects of distance to water on ant taxonomic richness, and a positive association between distance to water and ant taxonomic alpha diversity during the wet season. We suggest that at least part of this spatial pattern was caused by a trampling‐mediated effect on plant communities that often occurs around water sources. However, although there generally was higher ant abundance and diversity in the wet season, our results did not show that the effects of distance to water were higher in the dry than in the wet season. In addition, beta diversity was as high between‐ as within transects, and we did not find uniform support for nested community compositions along distance to water gradients. Instead, ant communities were mostly structured in a modular pattern. We interpret these results as strong effects of small‐scale local variation in vegetation characteristics on community structure.

## CONFLICT OF INTEREST

None declared.

## AUTHOR CONTRIBUTION

FD, TR, and BJR conceived the study; TR and CPH conducted field data collection; FD and TR conducted data analyses; and all authors contributed to writing the manuscript.

## DATA ACCESSIBILITY

Raw trap data as well as the spatial coordinates of the trapping locations are available in datadryad (https://datadryad.org), https://doi.org/10.5061/dryad.q7j256r.
