# Supplementary material for: The influence of distance to perennial surface water on ant communities in Mopane woodlands, northern Botswana
Source: Ecol Evol. 2018 Dec 27;9(1):154–65. doi: 10.1002/ece3.4692 (PMC6342134; doi:10.1002/ece3.4692)
Supplement: Supplementary file 1 [file ECE3-9-154-s001.doc]

**Appendix S1.** Supporting tables and figures.

**Table A1.** Abundances of each of the 29 ant genera caught using pitfall traps during the wet and dry season in semi-arid savanna Mopane woodlands in the Linyanti concession of northern Botswana. Genera are arranged alphabetically according to subfamily.

| Subfamily | Genus | Wet Season | Dry Season |
| --- | --- | --- | --- |
| Dorylinae | Aenictus | 8 | 1 |
| Dolichoderinae | Tapinoma | 595 | 109 |
| Dolichoderinae | Technomyrmex | 9 | 95 |
| Dorylinae | Dorylus | 27 | 11 |
| Formicinae | Anoplolepis | 138 | 0 |
| Formicinae | Camponotus | 221 | 179 |
| Formicinae | Lepisiota | 361 | 327 |
| Formicinae | Plagiolepis | 153 | 93 |
| Formicinae | Polyrhachis | 1 | 0 |
| Formicinae | Tapinolepis | 2 | 3 |
| Myrmicinae | Cardiocondyla | 411 | 153 |
| Myrmicinae | Carebara | 69 | 5 |
| Myrmicinae | Cataulacus | 0 | 1 |
| Myrmicinae | Crematogaster | 3 | 5 |
| Myrmicinae | Melissotarsus | 1 | 0 |
| Myrmicinae | Meranoplus | 110 | 6 |
| Myrmicinae | Messor | 18 | 3 |
| Myrmicinae | Monomorium | 8,740 | 2,561 |
| Myrmicinae | Myrmicaria | 2 | 1 |
| Myrmicinae | Ocymyrmex | 191 | 49 |
| Myrmicinae | Pheidole | 27,875 | 6,341 |
| Myrmicinae | Strumigenys | 24 | 1 |
| Myrmicinae | Solenopsis | 164 | 49 |
| Myrmicinae | Tetramorium | 3,061 | 1,572 |
| Ponerinae | Anochetus | 12 | 4 |
| Ponerinae | Leptogenys | 1 | 0 |
| Ponerinae | Pachycondyla | 1,047 | 217 |
| Ponerinae | Platythyrea | 1 | 0 |
| Pseudomyrmecinae | Tetraponera | 1 | 0 |
|  | TOTAL | 43,246 | 11,786 |

**Fig. A1.** Graphic representation of spatial nestedness for each of the six transects during both the wet and dry season in the Linyanti concession of northern Botswana. Transect 1 (a); transect 2 (b); transect 3 (c); transect 4 (d); transect 5 (e); transect 6 (f).

a)

WET

DRY

b)

DRY

WET

d)

c)

WET

WET

DRY

DRY

f)

e)

WET

WET

DRY

DRY

Appendix S2. Figures showing patterns of nestedness for each of the six transects during both the wet and dry season respectively in semi-arid savanna Mopane woodlands in the Linyanti concession of northern Botswana. Plots are created whereby distance categories are nested within the left-most distance category. Transect 1 (a); transect 2 (b); transect 3 (c); transect 4 (d); transect 5 (e); transect 6 (f).

**Fig. A2.** Graphic representation of modularity for each of the six transects during both the wet and dry season in the Linyanti concession of northern Botswana. Transect 1 (a); transect 2 (b); transect 3 (c); transect 4 (d); transect 5 (e); transect 6 (f).

a)

DRY

WET

WET

DRY

b)

c)

WET

d)

DRY

WET

DRY

DRY

WET

f)

DRY

WET

e)
